# Large variation in anti-factor Xa levels with nadroparin as thromboprophylaxis in COVID-19 and non-COVID-19 critically ill patients

**DOI:** 10.1186/s40360-024-00733-x

**Published:** 2024-02-06

**Authors:** Monique M.R. de Maat, Henk J. van Leeuwen, Lian Roovers, Sabine J.G.M. Ahlers, Jolanda Lambers, Marcel M.C. Hovens

**Affiliations:** 1 Department of Clinical Pharmacy, Rijnstate Hospital, Arnhem, The Netherlands; 2 Department of Internal Medicine and Intensive Care, Rijnstate Hospital, Arnhem, The Netherlands; 3 Department of Epidemiology, Rijnstate Hospital, Arnhem, The Netherlands; 4 Department of Clinical Chemistry and Hematology Laboratory, Rijnstate Hospital, Arnhem, The Netherlands; 5 Department of Internal Medicine, Rijnstate Hospital, Arnhem, The Netherlands

**Keywords:** Nadroparin, Thromboprophylaxis, Critically ill ICU patients, LMWH, COVID-19

## Abstract

**Purpose:**

Critically ill COVID-19 and non-COVID-19 patients receive thromboprophylaxis with the LMWH nadroparin. Whether a standard dosage is adequate in attaining the target anti-FXa levels (0.20–0.50 IU/ml) in these groups is unknown.

**Methods:**

This study was a prospective, observational study in the ICU of a large general teaching hospital in the Netherlands. COVID-19 and non-COVID-19 patients admitted to the ICU who received LMWH in a prophylactic dosage of 2850 IU, 5700 IU or 11400 IU subcutaneously were eligible for the study. Anti-FXa levels were determined 4 h after administration. Relevant laboratory parameters, prespecified co-variates and clinical data were extracted from the electronic health record system. The primary goal was to evaluate anti-FXa levels in critically ill patients on a prophylactic dosage of nadroparin. The second goal was to investigate whether covariates had an influence on anti-FXa levels.

**Results:**

A total of 62 patients were included in the analysis. In the COVID-19 group and non-COVID-19 group, 29 (96%) and 12 patients (38%) reached anti-FXa levels above 0.20 IU/ml, respectively. In the non-COVID-19 group, 63% of the patients had anti-FXA levels below the target range. When adjusted for nadroparin dosage a significant relation was found between body weight and the anti-FXa level (*p* = 0.013).

**Conclusion:**

A standard nadroparin dosage of 2850 IU sc in the critically ill patient is not sufficient to attain target anti-FXa levels in the majority of the studied patient group. We suggest a standard higher dosage in combination with body-weight dependent dosing as it leads to better exposure to nadroparin.

**Clinical trials registration:**

Retrospectively registered, ClinicalTrials.gov ID NTC 05926518 g, date of registration 06/01/23, unique ID 2020/1725.

## Introduction

Coronavirus disease 2019 (COVID-19) has spread rapidly around the globe after it was first recognized in Wuhan, China, in December 2019. While the pathophysiology underlying severe COVID-19 was then incompletely understood, a principal feature was a predominantly pro-thrombotic derangement of the haemostatic system [1]. The cumulative incidence of the composite outcome of symptomatic acute pulmonary embolism (PE), deep-vein thrombosis (DVT), ischemic stroke, myocardial infarction or systemic arterial embolism in the COVID-19 patient population on the Intensive Care Unit (ICU) was reported to be 31% (95% CI 20–41%) in 3 Dutch hospitals while standard thromboprophylaxis was administered [2]. This was much higher than the VTE incidence in historical patients with similar severity scores. Based on this observation, the Dutch guideline “COVID-19 coagulopathy” recommended to double the standard dose of thrombosis prophylaxis for COVID-19 patients when admitted to the Intensive Care Unit (ICU) [3].

Additional recommendations in the Dutch guideline were to increase the dose further in case of high body weight (> 100 kg) or decrease the dose in case of severe renal failure. At that time, evidence for these recommendations was mostly theoretical and not based on research. Monitoring of anti-Factor Xa (anti-FXa) levels was described in this guideline as optional in selected cases [3].

Anti-FXa levels are routinely monitored in our hospital in case of treatment with a low molecular weight heparin (LMWH) in a therapeutic dosage combined with risk factors for under- or overdosage, namely morbid obesity, pregnancy and severe renal failure. The established therapeutic range for peak anti-FXa levels (obtained 4 h after administration) is 0.6-1.0 IU/ml when nadroparin is dosed twice daily [4]. Anti-FXa activity is not routinely monitored when nadroparin is used in a prophylactic regimen. A reasonable accepted anti-FXa concentration in prophylactic regimens is 0.2–0.5 IU/mL 4 h post subcutaneous injection [5].

The standard dose that is used for thromboprophylaxis in the ICU in our hospital is nadroparin 2850 IU once daily (OD) subcutaneously (sc) for a regular ICU patient (non-COVID). For a COVID-19 patient the dosage advice of the Dutch guideline was implemented yielding nadroparin 5700 IU sc OD for a patient with a body weight less than 100 kg or 5700 IU twice daily (BID) sc for patients with a body weight of 100 kg and more. Vlot et al. reported a median anti-FXa activity in a cohort of 16 ICU COVID patients of 0.38 IU/ml when a standard dosage of 5700 IU sc BID was used for all COVID-19 patients regardless of weight [6]. It was at that moment not known whether nadroparin in a dosage based on body weight was adequate in light of achieving the accepted anti-FXa concentration of at least 0.2–0.5 IU/ml. It was also not known whether there was a difference in anti-FXa levels achieved in the COVID-19 ICU patient compared to the non-COVID-19 ICU patient, in which a lower dosage of nadroparin was administered.

Therefore, the primary goal of this study was to evaluate anti-FXa levels in COVID-19 and non-COVID-19 patients admitted to the ICU who were treated with a prophylactic dosage of nadroparin. Additionally, it was investigated whether covariates had an influence on anti-FXa levels.

## Methods

### Patients inclusion and data collection

This study was a prospective, single center, observational study in the ICU of a large general teaching hospital in the Netherlands. This study aimed to include at least 30 patients in each group, COVID and non-COVID patients. Patients were eligible for inclusion when they were 18 years of age at admission, had a diagnosis of COVID-19 or were admitted to the ICU for another medical (non-COVID-19) diagnosis and were using nadroparin in a prophylactic dosage and the estimated length of stay on the ICU was at least four days. A prophylactic dosage for COVID-19 patients included 5700 IU OD sc if weight was less than 100 kg and 5700 IU BID sc when weight was 100 kg or more [3]. For non-COVID-19 patients a prophylactic dosage included 2850 IU OD sc and 5700 IU OD sc in case of additional risk factors (including obesity) in the opinion of the attending physician. Patients were excluded when they had (a history of) Heparin Induced Thrombocytopenia and Thrombosis (HITT).

Anti-FXa activity was determined at least three days (peak level after fourth administration) after admission on the ICU. Patients could only be included once.

Relevant laboratory parameters and patient data were extracted from the hospital electronic health record system and transferred into a secured database. The following patient characteristics were collected for both groups at baseline: sex, age, body weight, length, C-reactive protein (CRP), Acute Physiology And Chronic Health Evaluation IV score (APACHE-IV), ICU indication in the non-COVID-19 group, medical history, renal function and coagulation parameters.

Clinical data that were collected included dosage of nadroparin, length of ICU stay, the occurrence of VTE or bleeding events during ICU admission and death during hospital admission. On the day of anti-FXa-level determination the following data were collected: use of vasopressor, cumulative fluid balance, CRP, renal function and use of renal replacement therapy.

Bleeding events were classified as major bleeding or clinically relevant non-major (CRNM) bleeding as previously published by the International Society of Thrombosis and Haematology (ISTH) [7–9]. Any bleeding not meeting the definitions of major or CRNM bleeding was classified as minor bleeding.

The study was approved by the medical research ethics committee of Rijnstate hospital. Patients or their legal representatives gave informed consent before inclusion in the study.

### Blood sampling and analytical assay

Blood sampling for anti-FXa activity was performed 3–5 h after subcutaneous dosing (peak anti-Xa level after fourth administration). Blood samples were collected in buffered 3.2% sodium citrate-containing tubes. All samples were centrifuged within 1 h after collection to obtain plasma samples. The plasma samples were stored at − 20˚C until analysis. Plasma concentrations of anti-FXa activity were measured with a STA-R Max3 (Diagnostic Stago, Asniere, France) using a chromogenic FXa inhibition assay (STA liquid anti-Xa, Diagnostic Stago, Asniere, France). The limit of detection of the assay was 0.1 IU/ml.

Target range peak anti-FXa concentrations for nadroparin in a prophylactic regimen was set at 0.2–0.5 IU/ml [5]. Other laboratory parameters were routinely obtained in the ICU and measured by the hospital clinical chemistry laboratory. Blood samples were taken from an indwelling arterial catheter.

### Statistical analysis

All data were analysed using SPSS statistical software (version 22.0.0.2). Patient characteristics on ICU admission, were collected for patients in the COVID-19 group and non-COVID-19 group. Differences between groups were studied using χ^2^ or Fisher’s exact tests for categorical data. The Mann–Whitney U test was used for continuous data. Linear regression was used to test whether there was a relation of co-variates with the anti-FXa level. The baseline co-variates nadroparin dosage, gender, age, weight, BMI, COVID-19 status, APACHE IV score and the covariates determined at day of anti-FXa-level vasopressor administration, renal function (eGFR = Estimated Glomerular Filtration Rate calculated with the Chronic Kidney Disease Epidemiology Collaboration (eGFR CKD-EPI)), cumulative fluid balance and CRP were tested for a relation with the anti-FXa-level. A p-value of < 0.05 was considered statistically significant.

## Results

### Characteristics of the patients

Between November 3, 2020, and February 16, 2022, a total of 70 patients were eligible for inclusion in this observational study. Of these 70 patients, eight patients could not be included in the analysis, because blood sampling was not done at steady state (four patients), patients were switched to therapeutic nadroparin before day of blood sampling (two patients), nadoparin administration was omitted at day of sampling (one patient) or because of technical problems with the analysis of the blood sample (one patient), resulting in a total of 62 patients in the study population.

As shown in Table 1, the study population had a median (interquartile [IQR]) age of 66 (59–72) years, 37 patients (60%) were men, and the median (IQR) body mass index (BMI) was 27.8 (24.3–32.0). In this study population, baseline characteristics of patients admitted to the ICU with the diagnosis COVID-19 were different from patients that were admitted to the ICU with an other diagnosis. COVID-19 patients had a significantly higher BMI (*p* = 0.018), a significantly higher eGFR (*p* = 0.036) and a significantly lower APACHE IV score (*p* < 0.001).

**Table 1:** Baseline patient characteristics

*Characteristic*	*Total* *n = 62*	*COVID-19* *n = 30*	*Non-COVID-19* *n = 32*
Male sex (number, %)	37 (60%)	20 (67%)	17 (53%)
Age (years)	66 (59–72)	66 (59–73)	67 (57–71)
Length (cm)^a^	173 (169–178)	173 (169–179)	173 (169–178)
Weight (kg)	85 (70–105)	91 (76–109)	83 (68–94)
BMI (kg/m^2^)^a^	27.8 (24.3–32.0)	30.0 (25.6–35.6)	26.6 (22.2–29.7)
APACHE IV score	72 (59–90)	65 (56–74)	89 (69–103)
Diagnosis (number, %)			
Pulmonary	41 (66%)		11 (35%)
of which COVID-19	30 (48%)	30 (100%)	
Cardiovascular	7 (11%)		7 (22%)
Renal	3 (4.8%)		3 (9%)
Gastro-intestinal	2 (3.2%)		2 (6%)
Other	9 (15%)		9 (28%)
Comorbidity (number, %)			
Hypertension	29 (47%)	14 (47%)	15 (47%)
MI/other cardiac diseases	19 (31%)	12 (40%)	7 (22%)
Diabetes Mellitus	16 (26%)	5 (17%)	11 (34%)
COPD/Asthma	15 (24%)	5 (17%)	10 (31%)
Morbid obesity	6 (9.7%)	4 (13%)	2 (6%)
CVA	5 (8.1%)	1 (3%)	4 (13%)
Chronic renal failure	4 (6.5%)	2 (7%)	2 (6%)
Malignancy	3 (4.8%)	1 (3%)	2 (6%)
None	3 (4.8%)	2 (7%)	1 (3%)
VTE (DVT/PE)	1 (1.6%)	-	1 (3%)
Other	52 (84%)	23 (77%)	29 (91%)
**Laboratory values at baseline** ^**b**^			
Creatinine (µmol/L)	76 (60–98)	65 (52–90)	87 (62–129)
Creatinine clearance (eGFR CKD-EPI, ml/min/1.73m^2^)	88 (64–98)	92 (70–102)	78 (43–95)
CRP (mg/L)	73 (25–193)	83 (53–167)	39 (4-277)
Hb (g/dL)	13.54 (11.60-14.18)	13.05 (11.28–14.02)	13.70 (11.76–14.99)
Platelet count (x10^9^/L)	260 (181–344)	251 (178–372)	266 (181–339)
D-dimer^c^ (µg/L)	-	2540 (928–5745)	-

Data are medians with interquartile ranges in parentheses unless otherwise stated

APACHE-IV = Acute Physiology And Chronic Health Evaluation-IV, BMI = Body Mass Index, COPD = Chronic Obstructive Pulmonary Disease, COVID-19 = Corona Virus Infectious Disease-2019, CRP = C-reactive Protein, CVA = Cerebro Vascular Accident, DVT = Deep Vein Thrombosis, eGFR CKD-EPI = Estimated Glomerular Filtration Rate calculated with the Chronic Kidney Disease Epidemiology Collaboration, Hb = Hemoglobin, MI = Myocardial Infarction, PE = Pulmonary Embolism, VTE = Venous ThromboEmbolism,

^a^3 missing data

^b^Normal ranges of measured laboratory tests were defined as follows:

53–97 µmol/L for creatinine, > 90 ml/min/1.73m^2^ for estimated Glomerular Filtration Rate calculated with the Chronic Kidney Disease Epidemiology Collaboration (eGFR CKD-EPI), < 4 mg/L for C-reactive protein (CRP), 11.92–15.95 mmol/L for women and 13.54–17.40 for men for hemoglobin (Hb) level, 150-400x10^9^/L for platelet count, and < 500 µg/L for D-dimer level.

^c^only 2 values of non-COVID-19 patients, 29 values of COVID-19 patient available

The most frequent comorbid diseases in both groups, non-COVID-19 and COVID-19 patients, included hypertension, diabetes mellitus, Chronic Obstructive Pulmonary Disease (COPD)/asthma and myocardial infarction (MI)/other cardiac disease.

### Anti-FXa levels

The anti-FXa levels corresponding with the different dosage regimes are presented in Table 2. All patients had dosages according to the national guidelines, no dosage reduction was performed in patients with kidney dysfunction. Of the 30 COVID-19 patients, 29 patients (96%) reached anti-FXA levels above 0.20 IU/ml 4 h after administration of nadroparin in the appropriate dosage. Only 12 (38%) non-COVID-19 patients reached anti-FXa levels in the target range of 0.20–0.50 IU/ml. In this non-COVID-19 group (*n* = 32) anti-FXa levels of 20 (63%) patients were below the target range, of which 11 were below the detection range of the analysis.

**Table 2:** Prophylactic nadroparin dosage and anti-FXa levels

*Characteristic*	*Total* *n = 62*	*COVID-19* *n = 30*	*Non-COVID-19* *n = 32*
LMWH dose			
2850 IU OD	30 (48%)	-	30 (94%)
5700 IU OD	21 (34%)	19 (63%)	2 (6%)
5700 IU BID	11 (18%)	11 (37%)	-
Anti-FXa level (IU/ml)			
Total	0.24 (0.11-0.33)	0.32 (0.24-0.56)	0.12 (0.00-0.25)
2850 IU OD	-	-	0.11 (0.00-0.24)
5700 IU OD	0.28 (0.23-0.44)	0.27 (0.23–0.48)	0.31 (0.30-0.31)^a^
5700 IU BID	-	0.52 (0.34–0.88)	-
Category Anti-FXa level IU/ml)			
0.20–0.50	31 (50%)	19 (63%)	12 (38%)
> 0.50	10 (16%)	10 (33%)	-
< 0.20	21 (34%)	1 (3%)	20 (63%)
< 0.10 (undetectable)	11 (18%)	-	11 (34%)

Data are medians with interquartile ranges in parentheses unless otherwise stated

Anti-FXa-levels = Anti-Factor Xa-levels, BID = twice daily, COVID-19 = Corona Virus Infectious Disease-2019, IU = International Unit, OD = once daily

Values above 0.50 IU/ml were in the range of 0.51–1.20 IU/ml of which 5 were above 0.60 IU/ml. Those 5 anti-FXa levels are considered in the therapeutic range of nadroparin treatment.

^a^two patients of which anti-FXa level was 0.30 IU/ml and 0.31 IU/ml

### Clinical outcome

Table 3 presents the clinical events bleeding and/or development of a VTE during ICU admission and in-hospital death. Overall, 14 patients experienced 20 bleeding events, of which six were bleeding events in the category major bleeding. Eleven of the 14 patients (six COVID and five non-COVID patients) had 16 bleeding events during a prophylactic dosage of the LMWH. Three COVID patients were switched to a therapeutic dosag of the LMWH because of development of a pulmonary embolism (PE) during IC admission and had a bleeding event during therapeutic dosage.

**Table 3:** Clinical events bleeding, VTE or death

	Number of patients (number of events or %)	COVID (*n* = 30) (number of events or %)	Non-COVID (*n* = 32) (number of events or %)
**Bleeding**			
**Total**	14 (20)	9 (12)	5 (8)
**Major**	3 (6)	1 (1)	2 (5)
**CRNM**	5 (6)	3 (4)	2 (2)
**Minor**	6 (8)	5 (7)	1 (1)
**Venous TromboEmbolism (VTE)**			
**Total (%)**	7 (11%)	7 (23%)	0 (0%)
DVT	1	1	0
PE	6	6	0
**Death**			
**Total (%)**	12 (19%)	5 (17%)	7 (22%)

COVID = Corona Virus Infectious Disease, CRNM = Clinically Relevant Non-Major bleeding, DVT = Deep Vein Thrombosis, PE = Pulmonary Embolism, VTE = Venous TromboEmbolism

Bleeding event or VTE during IC stay, death during hospital stay

None of the non-COVID patients developed a VTE during ICU admission, whereas in the COVID-group one patient suffered from DVT and six patients developed a PE. Of the 62 patients, almost 20% died during hospital stay.

#### Relation covariates and anti-FXa levels

**Fig. 1:**
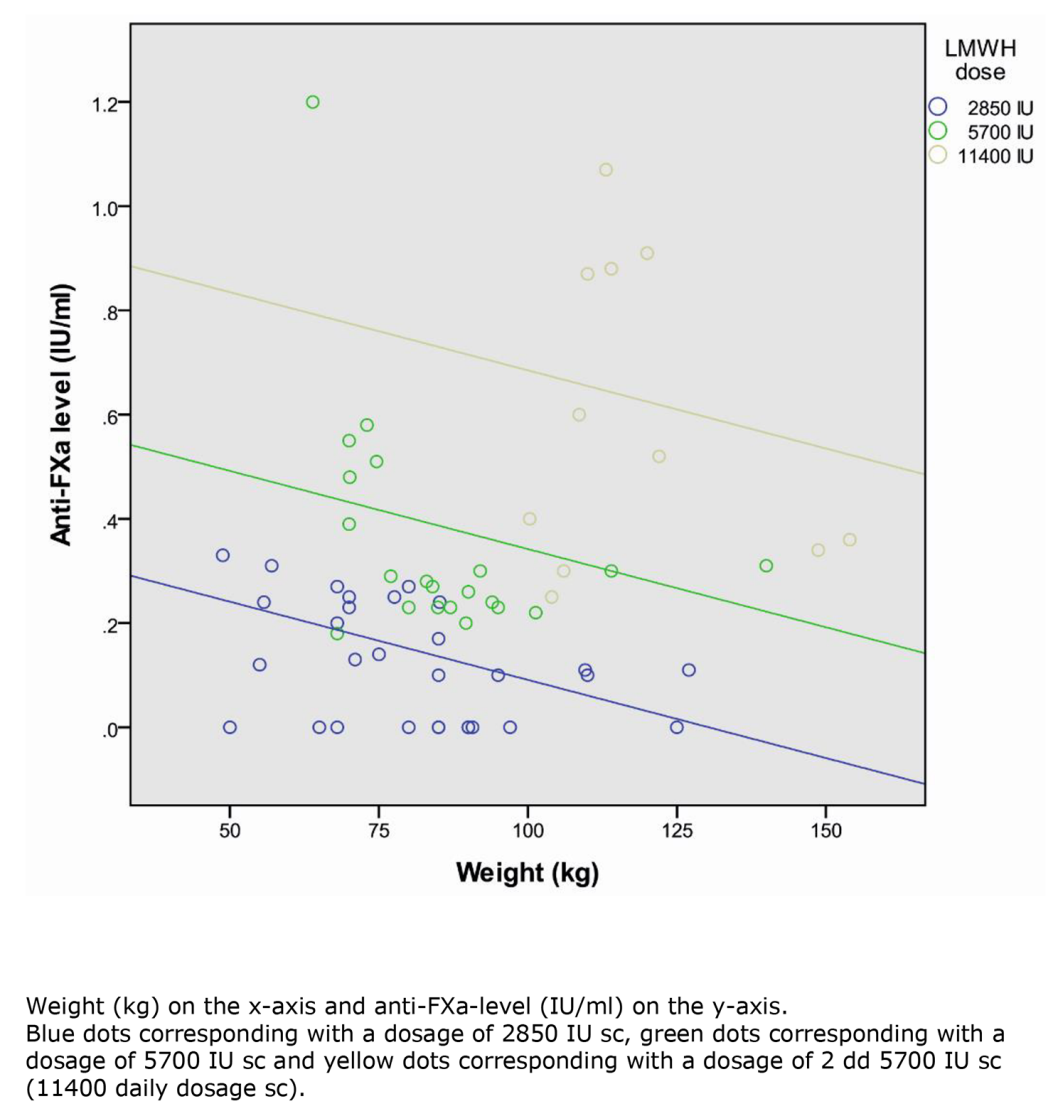
Weight versus anti-FXa levels with three thromboprophylactic dosages of nadroparin

The nadroparin daily dosage was significantly related with the anti-FXa level (Regression Coefficient 0.23 [0.12–0.35] for the 5700 IU dosage and 0.47 [0.33–0.61] for the 11400 IU dosage) in relation to the 2850 IU dosage (*p* < 0.001 for both dosages) (Table 4). When adjusted for daily nadroparin dosage a significant relation was found between body weight and the anti-FXa level (*p* = 0.013) For every increase in body weight of 10 kg the anti-FXa level reduced with 0.03 IU/ml. No significant correlation was found with other covariates (Fig. 1; Table 4).

**Table 4:** Covariate regression analysis of factors related to anti-FXa levels

Parameter	Regression coefficient (95% coefficient interval)	p-value
2850 IU	0.12 (0.05–0.19)	0.001
5700 IU*	+ 0.23 (0.12–0.35)	< 0.001
11400 IU*	+ 0.469 (0.331–0.606)	< 0.001
Baseline parameter		
Gender^a^	-0.07 (-0.17–0.03)	0.151
Age (years)^b^	0.001 (-0.004–0.007)	0.601
Weight (kg)^b^	-0.003 (-0.006– -0.001)	0.013
BMI (kg/m^2^)^b^	-0.007 (-0.014–0.001)	0.083
COVID-19^a^	-0.06 (-0.35–0.24)	0.700
APACHE IV score ^b^	0.001 (-0.001–0.004)	0.210
Day of anti-FXa level		
Cumulative fluid balance^b^	8.95x10^− 6^ (-3.95x10^− 5^– 5.74 x 10^− 5^)	0.713
Vasopressor use^a^	-0.07 (-0.17–0.03)	0.173
eGFR^b^	-0.001 (-0.002–0.0001)	0.147
CRP^b^	0.000 (-0.001–0.000)	0.118

*in relation to the 2850 IU dosage

^a^Dichotomous variable, ^b^Continue variable

Anti-FXa level = anti-Factor Xa level, APACHE-IV = Acute Physiology And Chronic Health Evaluation-IV, BMI = Body Mass Index, COVID-19 = Corona Virus Infectious Disease-2019, CRP = C-reactive Protein, eGFR = Estimated Glomerular Filtration Rate, IU = International Unit

## Discussion

In this observational study, the peak anti-FXa levels are described in critically ill non-COVID-19 and COVID-19 patients treated with the registered nadroparin prophylactic dosage of 2850 IU OD sc and increased dosages of 5700 IU OD sc or BID sc in accordance with the then current Dutch guidelines for anticoagulation in COVID-19. The included population is representative for COVID-19 and medical non-COVID-19 patients that are admitted to the ICU in a large teaching hospital in the Netherlands.

A large variation in peak anti-FXa levels was found ranging from undetectable to 1.20 IU/ml after administration of 2850 IU to 11400 IU sc per day. Undetectable anti-FXa levels were only seen in the low dose group, 2850 IU (18%) whereas only one patient in the 5700 IU dose group had an anti-FXa level that was below the target level of 0.20 IU/ml. A higher dosage than 2850 IU sc daily in a subset of critically ill patients seems therefore more appropriate.

Nonetheless, no VTEs were observed during ICU admission in the 2850 IU (non-COVID-19) group in contrast to the COVID-19 group in which seven patients developed a VTE. The VTE incidence was 23% which was in line with earlier observations [2], despite an increased dose of thromboprophylaxis in the COVID-19 patient group. The conclusion that an increased dosage of the LMWH does not reduce the VTE incidence (and probably increases the incidence of bleeding events on the other side) was also drawn by the Dutch Federation of Medical Specialists which decided to adjust the Dutch guideline for anticoagulation of COVID-19 in May 2022 based on the published literature to date, i.e. equal thromboprophylaxis dose for every patient COVID-19 or non-COVID-19. Other pro-thrombotic mechanisms probably play a more significant role in the COVID-19 patient group [10].

The incidence of VTE in the non-COVID-19 group could be an underestimation. In contrast to the medical protocol for the COVID-19 patient, routine determination of D-dimers and a Computed Tomography Pulmonary Angiogram (CTPA) of the chest to exclude or diagnose a PE are not common practice in the non-COVID-19 ICU patient [11].

Nadroparin exhibits theoretically linear pharmacokinetics with proportionality between anti-FXa plasma concentration and dose [12]. This was confirmed in our study which showed a significant relation between dose and anti-FXa-level. Bioavailability after subcutaneous injection is more than 90%. The volume of distribution is approximately equal to the blood volume. The peak anti-FXa level is attained 3 to 6 h after administration. Elimination is mainly via the renal route with an apparent plasma elimination half life of 3.5 h [12]. We observed a large proportion of low or undetectable anti-FXa peak levels with dosages of 2850 IU nadroparin sc in this critically ill patient group. Several studies describe the same phenomenon with nadroparin in dosages of 2850 IU or 3800 IU [13–15] and with the LMWH dalteparin in dosages of 2500 IU or 5000 IU [16, 17]. Additionally, ICU patients seem to have lower anti-FXa activity after LMWH subcutaneous administration when compared to healty individuals [18] or medical patients in regular wards [19]. Possible explanations for this lower exposure include decreased systemic absorption by the frequently concomitantly used vasopressors [14–16] or existence of extensive peripheral edema or increased distribution volumes, both factors associated with critical illness. In contrast with this theory, we did not find a relation of vasopressor use or cumulative fluid balance with the anti-FXa-concentration. An explanation could be the small sample size and/or the relative low dose and short duration of noradrenaline administration, especially in the COVID-19 group.

When adjusted for nadroparin dosage, only weight was found to be significanty related to the anti-FXa level. This finding is described earlier in the literature, where weight or BMI was inversely related to the anti-FXa-level when a LMWH was given as thromboprophylaxis [13, 16, 19, 20].

Because low anti-FXa-levels are considered to increase the VTE risk in other patient populations [20], the question arises whether the registered dose of nadroparin 2850 IU once daily is adequate for the critically ill medical patient population. Additionally, since body weight appears to influence the exposure to nadroparin, a combination of weight adjusted dosing followed by measurement of anti-FXa levels could lead to reduced risk for VTE. Wu et al. showed in their meta-analysis that patients on LMWH thromboprophylaxis could benefit from anti-FXa monitoring [21].

We suggest a standard higher dosage in combination with body-weight dependent dosing in ICU patients as it could lead to better exposure to nadroparin. A prospective study comparing body weight adjusted dosing of nadroparin with or without determination of anti-FXa-levels on clinical outcome in critically ill patients would be necessary to definitely answer this question.

## Data Availability

The datasets generated during and/or analysed during the current study are available from the corresponding author on reasonable request.
